# The Monitoring of Physical Health Observations After the Administration of Rapid Tranquillisation

**DOI:** 10.1192/bjo.2025.10692

**Published:** 2025-06-20

**Authors:** Viktoria Turimatsova

**Affiliations:** Royal Stoke University Hospital, Stoke-on-Trent, United Kingdom

## Abstract

**Aims:** Rapid tranquillisation is a restrictive practice used to manage acute behavioural disturbance, where medication is given in the form of an IM injection. The first-line medication used is lorazepam. There is an increased risk of the emergence of serious side effects (sedation, loss of consciousness and respiratory depression/arrest) from giving lorazepam via the IM route. MPFT SOP states that physical observations must be checked at a specified frequency and duration and recorded on the restrictive interventions monitoring form found on the RIO IT system. The monitoring at Norbury House (PICU) in Stafford (MPFT) is often incomplete. This audit evaluates the current adherence to the SOP by reviewing the monitoring of physical observations after the administration of rapid tranquillisation, identifying some of the reasons for incomplete monitoring and areas of practice that require improvement. This audit aims to demonstrate the importance of physical health monitoring and focus on improving patient safety by ensuring stricter adherence to monitoring protocols.

**Methods:** Data was collected between 8 September and 8 November 2024. To assess the current compliance with the SOP, data will be collected from the EPMA and RIO IT systems to check that the physical observations have been recorded at the correct frequency and duration as per SOP. To identify some of the reasons for incomplete monitoring, a Microsoft form questionnaire will be sent to staff members at Norbury to complete anonymously. The collected data will be used to identify areas of practice that require improvement.

**Results:** From twenty-one cases, there was one case where monitoring was completed, five cases where no monitoring or documentation was recorded, eleven cases where monitoring and documentation were recorded but not completed and four cases where monitoring and documentation were partially completed. Based on the eleven questionnaire responses, three responses outlined the SOP correctly, four were unsure, and the remaining four were incorrect. Barriers to completing monitoring included patient agitation, time restrictions, forgetting to document, no computer access and low staffing levels. Suggestions for support included education, appropriate delegation of tasks, EPMA alerts, adequate staffing levels and frequent re-auditing.

**Conclusion:** There is evidence that the current adherence to monitoring protocols is below the set standard. The data collected demonstrates that monitoring is often incomplete. The questionnaire responses highlighted the gaps in knowledge of the SOP and the existing barriers to completing the monitoring. Measures that could be taken may include staff education, alerts and frequent re-auditing.

